# The N-terminal Acetylation of α-Synuclein Changes the Affinity for Lipid Membranes but not the Structural Properties of the Bound State

**DOI:** 10.1038/s41598-019-57023-4

**Published:** 2020-01-14

**Authors:** Matteo Runfola, Alfonso De Simone, Michele Vendruscolo, Christopher M. Dobson, Giuliana Fusco

**Affiliations:** 1 Department of Life Sciences, Imperial College London, SW7 2AZ London, UK; 2 Department of Chemistry, University of Cambridge, Cambridge, CB2 1EW UK

**Keywords:** Protein aggregation, NMR spectroscopy

## Abstract

The aggregation of α-synuclein (αS), a protein abundant at presynaptic terminals, is associated with a range of highly debilitating neurodegenerative conditions, including Parkinson’s disease (PD), dementia with Lewy bodies (DLB) and multiple system atrophy (MSA). Emerging evidence indicates that the interaction of αS with lipid membranes defines both its physiological function and pathological effects. The characterisation of the modes of membrane binding by αS is therefore crucial to clarify the balance between normal and aberrant behaviour of this protein. Here we used solid-state nuclear magnetic resonance (ssNMR) spectroscopy to probe the nature of the N-terminally acetylated form of αS (NTAc-αS) bound to synaptic-like lipid vesicles. This post-translational modification is prevalent for the physiological form of αS and modulates the binding to lipid bilayers. By probing the structure, dynamics and membrane topology of NTAc-αS, we found that N-terminal acetylation does not alter significantly the conformational and topological properties of the membrane-bound state of αS, despite increasing its propensity for binding. Taken together, our data and previous characterisations of the cytosolic state of NTAc-αS clarify that the role of the N-terminal acetylation is to regulate the binding affinity of αS for synaptic vesicles without altering the structural properties of the bound state.

## Introduction

α-Synuclein (αS) is a 14 kDa monomeric disordered protein preferentially found at pre-synaptic termini^1^. The aggregation of αS is strongly linked with Parkinson’s disease (PD)^2–6^, as its aggregates are the major constituents of Lewy bodies in PD patients^7–9^. Point mutations, as well as duplications and triplications, in the αS gene are also associated with familial forms of PD^10–15^, and fibrillar aggregates of the non-amyloid-β component (NAC) region of the protein (residues 61–95) are associated with Alzheimer’s disease^16^.

The function of αS is currently still debated. Several lines of evidence have indicated a possible role for this protein in the regulation of the trafficking of synaptic vesicles (SVs)^17^, including the maintenance of pools of SVs at the synaptic termini^18–22^, the promotion of interactions between SVs^23–26^ and the assistance of SNARE formation during neurotransmitter release^27–30^. αS has also been shown to bind mitochondrial membranes, where it may have role in the mitigation of oxidative stress of mitochondria^31–33^. A common characteristic of the majority of these putative functions is that they all require the binding of αS to lipid membranes^34^. Indeed, the partition between membrane-bound and cytosolic forms of αS is tightly regulated *in vivo*^35^ and appears to be crucial for the mechanism of αS aggregation^21,36–41^ as well as the toxicity of its fibrillar oligomers^42^. Understanding the biological regulation of this interaction is therefore a major priority to clarify the balance between functional and dysfunctional aspects in this presynaptic protein.

Upon membrane binding αS undergoes a transition from an intrinsically disordered monomeric protein^43–45^ to a conformation that is enriched in α-helical structure. This ordering process is promoted by 7 imperfect sequence repeats of 11 residues located in the first 90 residues of the protein sequence and encoding for amphipathic α-helical motifs^45^. The modular organization of the repeats provides αS with the ability to bind a variety of assemblies ranging from small detergent micelles to lipid vesicles and membranes^43,46,47^ using a multiplicity of distinct binding modes^43^.

Membrane binding by αS is modulated by some post-translational modifications (PTMs), including the phosphorylation of different residues in the sequence^48–50^. Moreover, the N-terminal acetylation of αS^51,52^, which is found in both healthy individuals and PD patients, has been shown to induce stronger membrane affinity, as well as an increased propensity to adopt transient α-helical structures in the N-terminal region^53,54^, a property that is enhanced upon copper interaction^55,56^.

As the N-terminally acetylated αS (NTAc-αS) is considered to be the physiologically relevant form, and because of the central role of membrane binding for both functional and pathological contexts, it is important to investigate how this PTM alters the structure and dynamics of the membrane bound state of αS. Although previous studies have analysed the interaction between NTAc-αS and lipid membranes by probing the properties of its unbound state^50,53,54,57^, a full understanding of the structural and dynamical properties of the membrane-bound state of NTAc-αS is currently missing.

To answer to this key question, we here employed solid-state NMR (ssNMR) to directly probe the conformational properties of NTAc-αS at the surface of small unilamellar vesicles (SUVs) that mimic synaptic vesicles for composition and size^43,44^. Using an approach based on paramagnetic relaxation enhancement (PRE) effects, we investigated the impact of the N-terminal acetylation on the topological properties of the protein with respect to the lipid membrane. Our experiments elucidated the nature of the membrane-bound state of NTAc-αS and clarified further the role of this PTM on the regulation of the membrane interaction of αS.

## Results

### ssNMR spectra of NTAc-αS bound to lipid vesicles

**Figure 1:**
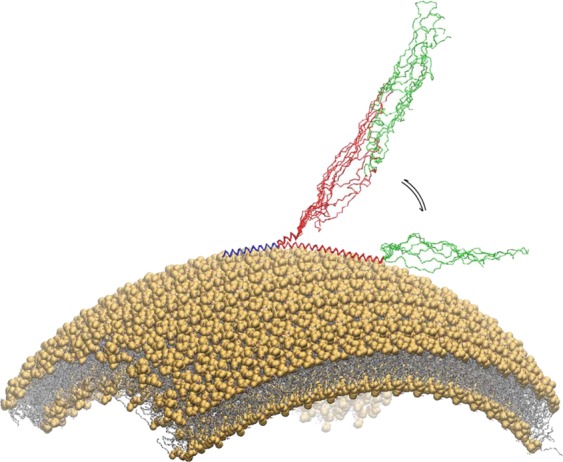
Representation of the SUV-bound state of αS. Schematic illustration of the binding mode of αS to a lipid vesicle. Three regions of protein have different dynamical and structural properties upon binding with the membrane^44^. The 25 N-terminal residues (blue) are rigidly anchored to the membrane surface, adopting a stable α-helical conformation. The central region, spanning residue 26–98 (red), is in equilibrium between bound-tethered and unbound-detached conformations. The C-terminal region (residues 98–140, green) remains unstructured and weakly associated with the membrane surface.

Previous magic angle spinning (MAS) ssNMR studies of non-acetylated αS identified three different regions of the protein having distinctive structural and dynamical properties in the membrane-bound state^44^. These regions include the N-terminal portion of the protein, which spans the initial 25 residues and acts as the primary membrane-binding motif that adopts the conformation of a stable amphipathic helix anchoring the protein onto the membrane surface. It was possible to assign the ssNMR resonances of the segment 6–25 of this region^44^ and employ these data to refine its structural ensemble when bound to synaptic-like vesicles^58^. In addition to the rigid N-terminal anchor, a central ‘sensor’ region (residues 26 to 98) resulted to have enhanced relaxation properties in the ssNMR spectra, suggesting that it exists in equilibrium between multiple conformations at the membrane surface (Fig. 1)^44^. The central region was shown to modulate the overall affinity of the protein for lipid bilayers^44^ as well as to be involved in the mechanism by which αS promotes the clustering of synaptic vesicles^23^. Finally, the C-terminal residues (99 to 140) were found to remain largely unstructured and unbound to lipid bilayers, with some residues establishing transient contacts with the membrane surface^44^.

**Figure 2:**
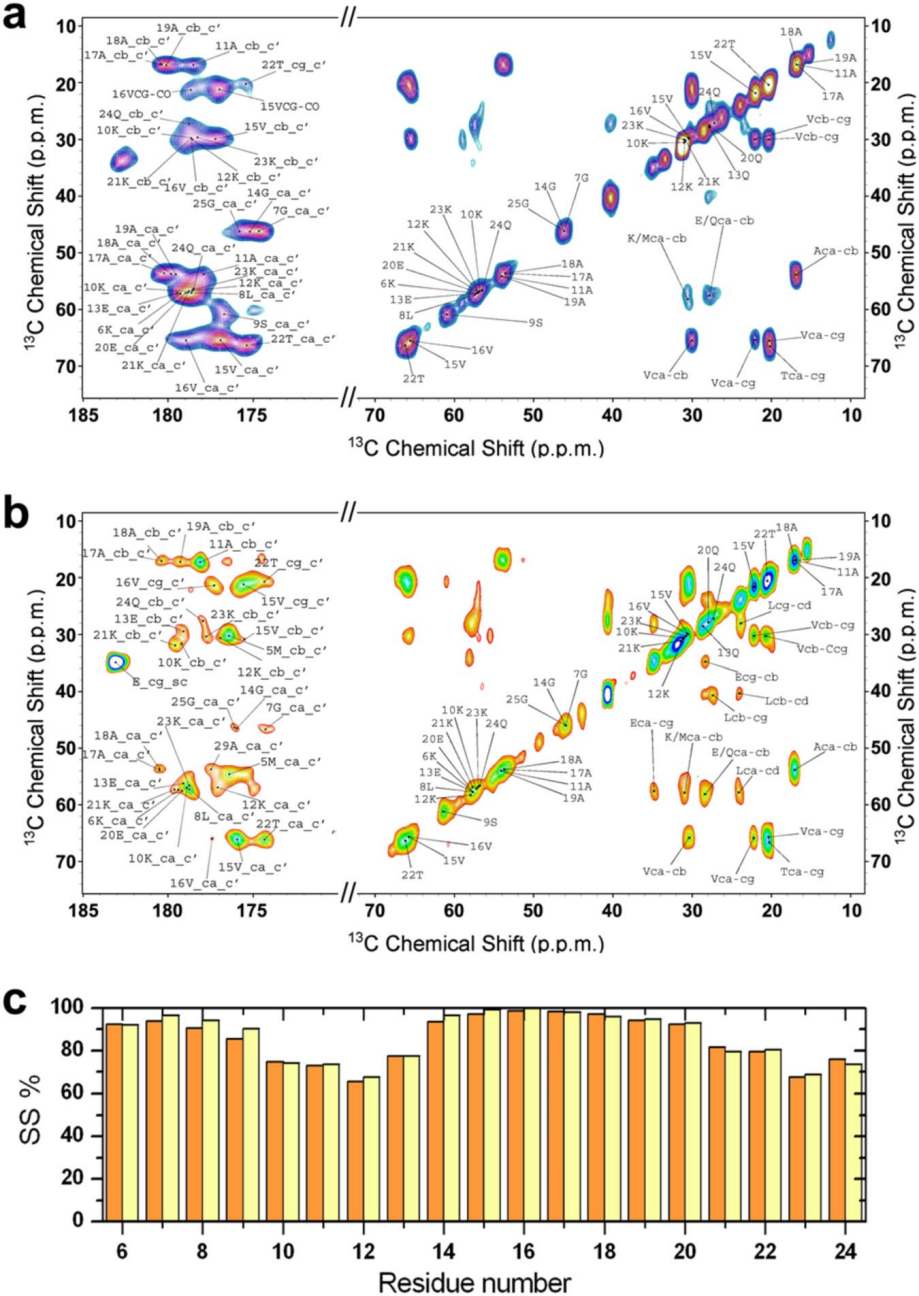
MAS ssNMR spectra to detect rigid regions of NTAc-αS bound to SUVs. (**a,b**) ^13^C-^13^C DARR correlation spectra were measured for NTAc-αS bound to DOPE:DOPS:DOPC (a) and POPG (b) vesicles using 1 ms and 50 ms for contact and mixing times, respectively. The spectra were recorded at −19 °C to favour the gel phase of the lipid membrane and obtain enhanced signal to noise measurements. The experiments were performed at a ^1^H Larmor frequency of 700 MHz using a 3.2 mm E^Free^ probe and a spinning rate of 10.0 kHz. Cross correlations between aliphatic-aliphatic and aliphatic-carbonyl regions are shown, with residue names reported with the single letter convention. Assignments of the spectra were derived from previous studies of vesicle bound αS^44^. Atom names ca, cb, cg, cd are used for C^α^, C^β^, C^γ^ and C^δ^ atoms, respectively. **c)** Population of α-helix (residue specific percentage of secondary structure SS%) derived by using the chemical shifts from the ssNMR spectra as input for the δ2D program^77^. Non-acetylated and acetylated αS bound to DOPE:DOPS:DOPC vesicles are shown in yellow and orange, respectively.

We here used ssNMR to investigate the effects of N-terminal acetylation to the membrane-bound form of αS. ^13^C-^15^N labelled NTAc-αS samples were mixed with DOPE:DOPS:DOPC (5:3:2 w/w) SUVs at a protein:lipid ratio of 1:65^44^ and pelleted via ultracentrifugation into 3.2 mm rotors for MAS ssNMR. ^13^C-^13^C dipolar assisted rotational resonance (DARR)^59^ spectra of membrane-bound NTAc-αS, which were measured at −19 °C to enhance the signal to noise under conditions where this lipid mixture adopts a gel phase^60^, revealed a number of resonances belonging to residues from the N-terminal region in a rigid bound state at the surface of the SUVs (Fig. 2a). A close inspection of the resonances from these spectra revealed no significant differences between the chemical shifts of the membrane bound states of NTAc-αS and non-acetylated αS^44^. Similarly, by analysing the bound state of NTAc-αS with SUVs composed of POPG lipids, which induce stronger membrane binding by αS^61^, we obtained ^13^C-^13^C-DARR spectra that are essentially indistinguishable from those measured in the case of non-acetylated αS^44^ (Fig. 2b).

**Figure 3:**
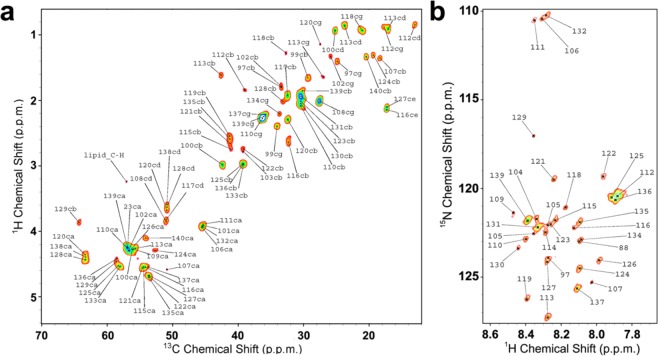
INEPT-based identification of dynamical regions of NTAc-αS bound to DOPE:DOPS:DOPC SUVs. ^13^C-detected ^1^H-^13^C correlation via INEPT transfer (**a**) and ^1^H-detected ^1^H-^15^N-HSQC (**b**) of NTAc-αS bound to DOPE:DOPS:DOPC SUVs recorded at 4 °C using MAS ssNMR with spinning rate of 10 kHz. The experiments were performed at a ^1^H Larmor frequency of 700 MHz using a 3.2 mm E^Free^ probe. Atom names ca, cb, cg, cd are used for C^α^, C^β^, C^γ^ and C^δ^ atoms, respectively. Assignment of the spectra were derived from previous studies of vesicle bound αS^44^.

In addition to ^13^C-^13^C-DARR spectra probing rigid regions of the protein, we measured insensitive nuclei enhanced by polarization transfer (INEPT) spectra^62^ to characterise the highly dynamic protein regions of NTAc-αS bound to DOPE:DOPS:DOPC SUVs (Fig. 3). ^13^C detected ^1^H-^13^C correlations and ^1^H detected ^1^H-^15^N correlations^63^ revealed a number of ssNMR resonances of residues in the disordered regions of the protein. The resulting chemical shifts closely match those measured in ^1^H-^13^C HSQC and ^1^H-^15^N HSQC spectra of the monomeric form of αS measured using solution NMR^44^. These spectra revealed the N-H and C-H cross peaks of residues spanning the region 97 to 140 of the protein, and showed no considerable differences with the chemical shifts measured using both monomeric-disordered αS in water and non-acetylated αS at the surface of SUVs.

Taken together, ssNMR experiments indicated that the dynamical and structural properties of both rigid and disordered regions of αS bound to the surface of lipid bilayers do not change significantly as a result of the N-terminal acetylation.

### Membrane insertion of the N-terminal anchor of NTAc-αS

**Figure 4:**
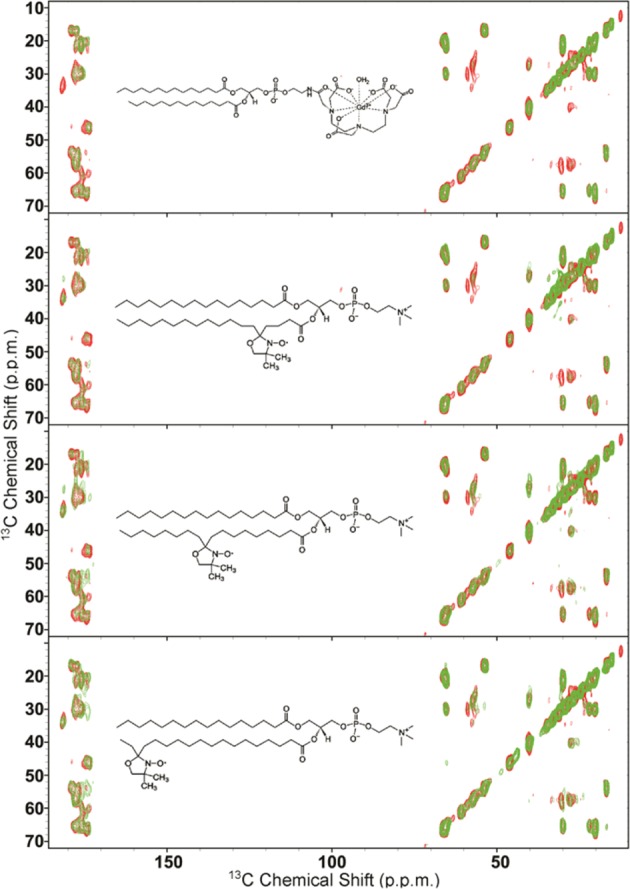
Membrane insertion properties of the N-terminal region of NTAc-αS using PREs. ssNMR PRE of membrane bound NTAc-αS were measured by doping to DOPE:DOPS:DOPC SUVs with 2% of lipid molecules having a paramagnetic centre in different positions ranging from the hydrophilic head group (1-palmitoyl-2-stearoyl-[5-doxyl]-sn-glycero-3-phosphocholine) to the position of carbons 5, 10 and 16 of the lipid tail (1-palmitoyl-2-stearoyl-[*N*-doxyl]-sn-glycero-3-phosphocholine, *N* = 5, 10, 16). The chemical structures of the paramagnetically labelled lipids are shown in the corresponding spectra. ^13^C-^13^C-DARR spectra of these samples were measured at −19 °C to favour the gel phase of the membrane^44^, and using 1 ms and 50 ms for contact and mixing times, respectively. The experiments were performed at a ^1^H Larmor frequency of 700 MHz using a 3.2 mm E^Free^ probe and a MAS rate of 10.0 kHz. Spectra in the presence and absence of the paramagnetic lipids are shown in green and red, respectively.

Next, we used ssNMR to directly probe the topological properties of the membrane-bound state of NTAc-αS. In particular, using paramagnetic relaxation enhancement (PRE)^64,65^ experiments, we could obtain detailed information of the levels of protein insertion into the hydrophobic region of the lipid bilayer. These experiments were performed by doping the DOPE:DOPS:DOPC lipid mixture with low quantities (2%) of lipids that incorporate a paramagnetic centre into their chemical structure. The spatial vicinity of protein regions to this centre enhances the transverse relaxation of their NMR signals, thereby providing a map of the contacts between the protein and the spin. Four different types of paramagnetic lipids were used to obtain a map of the contacts between the protein and spins placed at different positions of the membrane, i.e. from the hydrophilic head groups to the last carbon of the hydrophobic tail (Fig. 4). PRE measured via ^13^C-^13^C-DARR spectra by doping the membrane with a paramagnetic centre in head groups (gadolinium salt of PE-DTPA, Avanti Polar Lipids, USA) provided evidence of contacts between atoms of the N-terminal region of NTAc-αS and the membrane surface. The spectra showed selective paramagnetic broadening of ^13^C-^13^C DARR resonances for both aliphatic-aliphatic and aliphatic-carbonyl cross-peaks, and suggested the topology of an amphipathic helix laying parallel on the membrane surface (Fig. 4). We then probed the degree of insertion of the N-terminal region of NTAc-αS into the hydrophobic region of the membrane by measuring PREs with paramagnetic lipids having spins at the positions of carbons 5, 10 and 16 of the lipid tail. These PRE experiments showed a gradual reduction of the paramagnetic broadening effect when changing the position of the spin from carbon 5 to carbon 16 of the lipid tail (Fig. 4), with the latter showing no evidence of PRE effect in the DARR spectrum.

These data indicate mild levels of insertion of the N-terminal region of NTAc-αS into the lipid bilayer, a result that is consistent with PRE experiments previously performed under the same experimental conditions by using non-acetylated αS^44,58^.

## Discussion

Although close links between PD and the aggregation of αS are widely recognised, the physiological role of this presynaptic protein remains largely unclear^17^. It has now become evident that a fundamental element for the biological activity of αS is the interaction with lipid membranes^66^, which appears to be crucial in defining the balance between normal and aberrant forms of this protein. The membrane-binding affinity by αS is primarily influenced by the properties of the lipid bilayer, including curvature, charge, packing defects and surface hydrophobicity^46,47,67–69^, but other factors such as calcium binding^70^, phosphorylation^48–50^ and N-terminal acetylation^51,52^ have also been shown to modulate this interaction.

**Figure 5:**
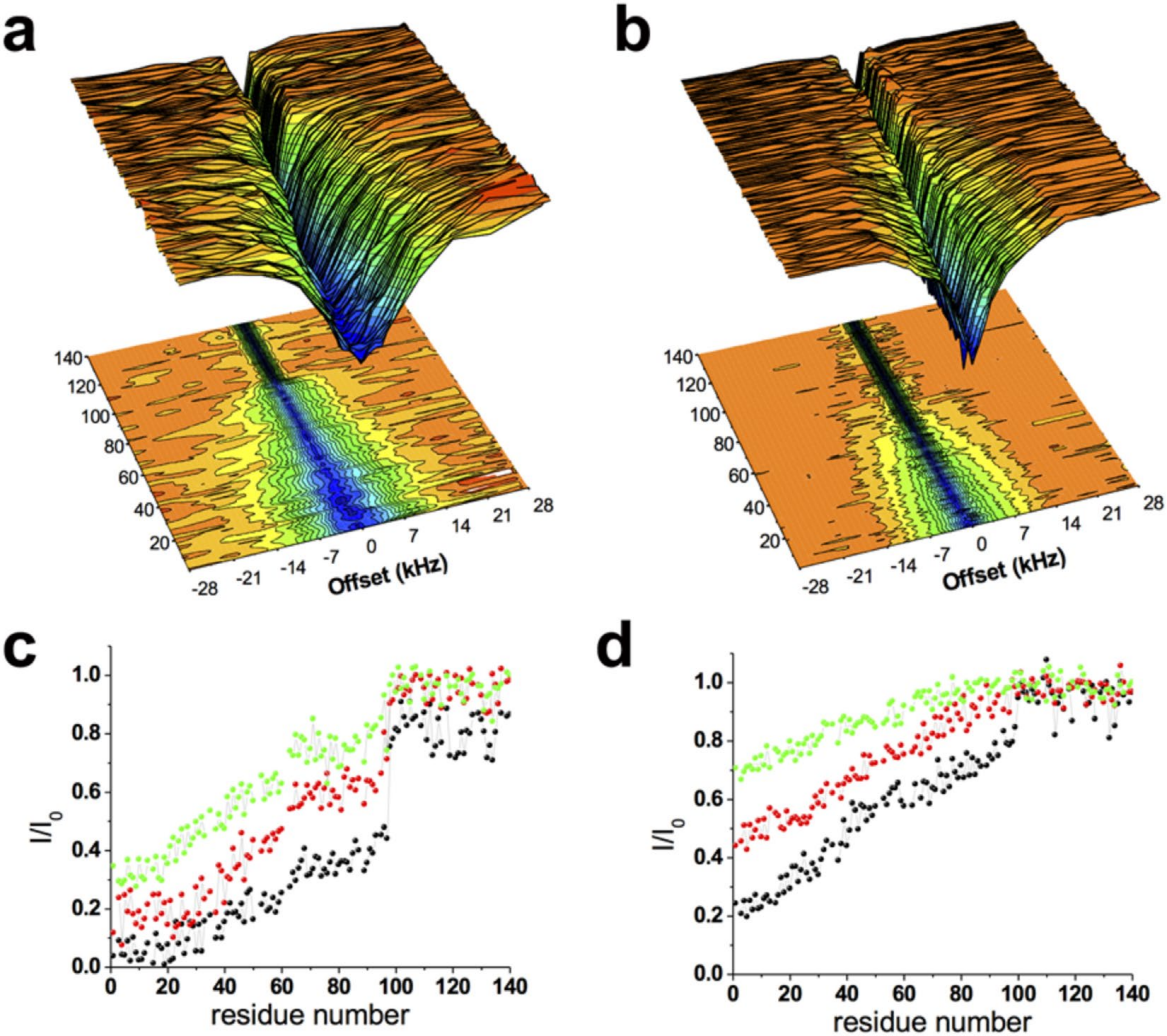
CEST experiments probing the binding equilibrium of NTAc-αS. CEST ^1^H-^15^N HSQC spectra were recorded by applying a continuous wave saturation (170 Hz or 350 Hz) on the ^15^N resonance with offsets ranging from −28 kHz to + 28 kHz, and using a reference spectrum measured with an offset of 100 kHz. Measurements were carried out using a protein concentration of 300 μM and 0.06% (0.6 mg/ml) of SUVs composed of DOPE:DOPS:DOPC lipids in a ratio of 5:3:2 (w/w). (**a,b)** CEST surfaces measured using bandwidths of 350 Hz and 170 Hz, respectively. (**c,d)** CEST saturation along the αS sequence measured using bandwidths of 350 Hz and 170 Hz, respectively. Black, red and green lines refer to the averaged CEST profiles measured using offsets at + /− 1.5 kHz, + /− 3 kHz and + /− 5 kHz, respectively.

We have used here ssNMR spectroscopy to investigate the structure, dynamics and membrane-topology of NTAc-αS bound to synaptic-like vesicles. The acetylation of the N-terminal residue of αS is indeed prevalent in both healthy individuals and PD patients^51–56^, and characterising its influence on the modes of membrane binding is crucial to understand biological behaviour of this protein. Previous solution NMR studies have probed this interaction by studying the properties of the unbound state of NTAc-αS in equilibrium with its membrane-bound form^53,54^. The resulting spectra showed increased relaxation properties for the resonances of the N-terminal region of the protein indicating stronger membrane-binding for this region when the protein is acetylated. This result is consistent with the present chemical exchange saturation transfer (CEST) experiments that enabled to characterise the equilibrium between bound and unbound states at a residue-specific level (Fig. 5). CEST measurements also showed no significant decrease in the saturation of residues that immediately follow the N-terminal region, as observed in the non-acetylated case^44^, indicating stronger membrane affinity for these residues in the case of NTAc-αS.

Despite these observations of higher membrane affinity in NTAc-αS, our ssNMR spectra indicate that this PTM does not alter the conformational properties of the membrane-bound state of the protein. In particular, cross polarisation and INEPT spectra, probing respectively rigid and highly dynamical regions of the protein (Figs. 2 and 3), indicated no substantial differences in the structure and dynamics of the membrane-bound states of NTAc-αS compared to non-acetylated αS^44^. The analysis of the chemical shifts of the bound states of NTAc-αS and non-acetylated αS showed a similar content of α-helix in the N-terminal region (Fig. 2c), indicating a single binding mode for this protein segment in both protein constructs. Moreover ssNMR PRE experiments, provided evidence that the modes of membrane insertion of the N-terminal anchor of αS are not significantly altered by the N-terminal acetylation.

Taken together, these data indicate that the enhanced affinity of NTAc-αS for binding biological membranes is not the result of any specific alteration of its membrane-bound state, and suggest that it is likely promoted by changes in the properties of the unbound αS, particularly the increased α-helical character in the N-terminal region^53^. The presence of transient α-helical conformations in the N-terminal region of the unbound NTAc-αS has the likely effect of relieving part of the entropic cost associated with the disorder-to-order transition required for the membrane binding. This intrinsic α-helical propensity may also favour the reversibility of the membrane interaction, which is essential to promote a rapid equilibrium between bound and unbound states, a key element for the ability of αS to facilitate the interaction between synaptic vesicles^23–26^. This biological property has been associated with the putative function of αS of regulating distal pools of synaptic vesicles that play a key role for the SV homeostasis during neurotransmitter release^20–22^.

In conclusion the topological and structural properties of the membrane-bound state of NTAc-αS that we have described here contribute to the definition of the mechanism by which the N-terminal acetylation regulates the mechanism of binding to synaptic membranes.

## Methods

### NTAc-αS purification

αS was expressed and purified as previously described^44^. Briefly the protein was expressed in *E. coli* using plasmid pT7–7^44^. In order to obtain N-terminal acetylation of αS we used coexpression with a plasmid carrying the components of the NatB complex (Addgene).

After transforming in BL21 (DE3)-gold cells (Agilent Technologies, Santa Clara, USA), uniformly ^15^N and/or ^13^C labeled αS variants were obtained by growing the bacteria in isotope-enriched M9 minimal media containing 1 g^.^L^−1^ of ^15^N ammonium chloride, 2 g^.^L^−1^ of ^13^C-glucose (Sigma-Aldrich, St Louis, USA) and 1 g of protonated IsoGro ^15^N-^13^C (Sigma, St. Louis, MO). The growth was obtained at 37 °C under constant shaking at 250 rpm and supplemented with 100 μg^.^ml^−1^ ampicillin to an OD600 of 0.6. The expression was induced with 1 mM isopropyl β-D-1-thiogalactopyranoside (IPTG) at 37 °C for 4 h, and the cells were harvested by centrifugation at 6200 *g* (Beckman Coulter, Brea, USA). The cell pellets were resuspended in lysis buffer (10 mM Tris-HCl pH 8, 1 mM EDTA and EDTA-free complete protease inhibitor cocktail tablets obtained from Roche, Basel, Switzerland) and lysed by sonication. The cell lysate was centrifuged at 22,000 *g* for 30 min to remove cell debris. In order to precipitate the heat-sensitive proteins, the supernatant was then heated for 20 min at 70 °C and centrifuged at 22,000 *g*. Subsequently streptomycin sulfate was added to the supernatant to a final concentration of 10 mg^.^ml^−1^ to stimulate DNA precipitation. The mixture was stirred for 15 min at 4 °C followed by centrifugation at 22,000 *g*. Then, ammonium sulfate was added to the supernatant to a concentration of 360 mg^.^ml^−1^ in order to precipitate the protein. The solution was stirred for 30 min at 4 °C and centrifuged again at 22,000 *g*. The resulting pellet was resuspended in 25 mM Tris-HCl, pH 7.7 and dialyzed against the same buffer in order to remove salts. The dialyzed solutions were then loaded onto an anion exchange column (26/10 Q sepharose high performance, GE Healthcare, Little Chalfont, UK) and eluted with a 0–1 M NaCl step gradient, and then further purified by loading onto a size exclusion column (Hiload 26/60 Superdex 75 preparation grade, GE Healthcare, Little Chalfont, UK). All the fractions containing the monomeric protein were pooled together and concentrated by using Vivaspin filter devices (Sartorius Stedim Biotech, Gottingen, Germany). The purity of the aliquots after each step was analyzed by SDS-PAGE and the protein concentration was determined from the absorbance at 275 nm using an extinction coefficient of 5600 M^−1^ cm^−1^. Mass spectrometry was used to assess that the level of N-terminal acetylation was complete.

### Preparation of SUVs

Small unilamellar vesicles (SUVs) containing DOPE:DOPS:DOPC at a ratio of 5:3:2 (w/w) or POPG (Avanti Polar Lipids Inc., Alabaster, USA) were prepared from chloroform solutions of the lipids as described previously^43,44^. Briefly, the lipid mixture was evaporated under a stream of nitrogen gas and then dried thoroughly under vacuum to yield a thin lipid film. The dried thin film was then re-hydrated by adding aqueous buffer (20 mM sodium phosphate, pH 6.0) at a concentration of 15 mg^.^ml^−1^ (1.5%) and subjected to vortex mixing. In all NMR experiments described in this paper SUVs were obtained by using several cycles of freeze-thawing and sonication until the mixture became clear^43,44^. For ssNMR studies αS was added to the SUV mixtures up to a molar ratio of 1:65 protein:lipid. The mixtures were then pelleted at 303,747 *g* for 30 min at 4 °C (Beckman Coulter Optima TLX Inc. Brea, USA) by using a TLA 100.3 rotor. Subsequently the SUV-αS samples were transferred into 3.2 mm Zirconia XC thin-walled MAS rotors for ssNMR experiments.

### Magic angle spinning (MAS) measurements

MAS ssNMR measurements were used to directly probe the resonances of NTAc-αS in the vesicle-bound state, which are inaccessible to solution-state NMR. MAS experiments were carried out on a 700 MHz Bruker Spectrometer with a 3.2 mm E^Free^ probe. Dipolar assisted rotational resonance (DARR) experiments^59^ were performed at a MAS rate of 10 kHz using a mixing time of 50 ms and a contact time of 1 ms. DARR spectra were acquired at −19 °C to favour signal to noise by inducing a gel phase of the lipid membranes. Previous studies showed that the chemical shifts of αS bound to DOPE:DOPS:DOPC SUVs at −19 °C and 4 °C are indistinguishable, ruling out conformational changes of the protein as a result of the different phases of the lipids^44^. Insensitive nuclei enhanced by polarization transfer (INEPT) spectra^62^ were measured at 4 °C using a MAS rate of 10 kHz. Pulse widths were 2.5 μs for ^1^H and 5.5 μs for ^13^C, and ^1^H TPPM decoupling was applied at ωRF/(2π) = 71.4–100 kHz^44^. ^1^H detected ^1^H-^15^N-HSQC of membrane bound NTAc-αS was measured as in Gopinath *et al*.^63^. Assignments of the resonances of NTAc-αS in DARR and INEPT spectra were derived from our previous study of αS bound to SUVs or unbound αS^44^.

### Chemical exchange saturation transfer (CEST) experiments

CEST measurements^44,71–75^ probed the equilibrium between membrane unbound and membrane bound states of αS *via* direct detection of saturation in the resonances of the unbound state. In studying αS-SUV interactions, CEST shows higher sensitivity than measurements based on the signal attenuation in HSQC spectra, which enables measurements at low lipid:protein ratios to minimise αS or lipid aggregation^44^. Moreover, CEST signals are directly sensitive to the interaction between αS and the membrane surface and minimise the interference from additional factors that can contribute to the transverse relaxation rates of the protein resonances^72–75^. Solution state NMR experiments were carried out at 10 °C on a sample composed of αS (300 μM) incubated with DOPE:DOPS:DOPC SUVs at a concentration of 0.6 mg/ml and using a Bruker spectrometer operating at ^1^H frequencies of 700 MHz equipped with triple resonance HCN cryo-probe. CEST experiments were based on ^1^H-^15^N HSQC spectra by applying constant wave saturation in the ^15^N channel. Assignment of the solution NMR resonances was obtained from our previous studies^44^ and controlled with a series of 3D spectra by following a published protocol^76^. Since we aimed at probing the exchange between monomeric αS (having sharp resonances) and αS bound to SUVs (having significantly broader resonances), a series of large offsets was employed (−28, −21, −14, −9, −5, −3, −1.5, 0, 1.5, 3, 5, 9, 14, 21 and 28 kHz), resulting in CEST profiles of symmetrical shapes^44,72,73^. An additional spectrum, saturated at −100 kHz, was recorded as a reference. The CEST experiments were measured using a data matrix consisting of 2048 (t_2_, ^1^H) × 220 (t_1_, ^15^N) complex points.
